# Pharmacokinetic Interaction Between Isavuconazole and Rifabutin in a Real‐World Setting

**DOI:** 10.1111/myc.70157

**Published:** 2026-01-23

**Authors:** Sunish Shah, Lloyd Clarke, Tiffany Lee, Leah Georgiades, Brandon J. Smith, Raman Venkataramanan, Ryan M. Rivosecchi

**Affiliations:** ^1^ Antibiotic Management Program University of Pittsburgh Medical Center Pittsburgh Pennsylvania USA; ^2^ Department of Pharmacy University of Pittsburgh Medical Center Pittsburgh Pennsylvania USA; ^3^ InsightRX San Francisco California USA; ^4^ Department of Medicine University of Pittsburgh Pittsburgh Pennsylvania USA; ^5^ Department of Pharmaceutical Sciences, School of Pharmacy University of Pittsburgh Pittsburgh Pennsylvania USA

**Keywords:** isavuconazole, rifabutin, rifampin, therapeutic drug monitoring

## Abstract

**Background:**

Since rifabutin has less severe drug interactions, it is preferred over rifampin when administered concomitantly with azole antifungals. However, limited data exist to evaluate this interaction.

**Methods:**

This was a single‐centre study of hospitalised patients who received concomitant isavuconazole and rifabutin prior to plasma isavuconazole therapeutic drug monitoring. Isavuconazole was administered at a standard dose of isavuconazonium sulphate 372 mg every 8 h for six doses followed by 372 mg every 24 h.

**Results:**

Of the seven patients included, the median age (range) was 53 years (33–67), 71% (5/7) were solid organ transplant recipients and no patients had underlying cirrhosis. The median (range) corrected 24‐h steady‐state isavuconazole was 2.7 mg/L (0.7–3.7) and 86% (6/7) had an isavuconazole level > 1 mg/L. The median (range) area under the curve (AUC 24), half‐life (*T*_½_) and clearance (Cl) were 85 mg/L h (33–112), 13.1 h (6.8–17.8) and 4 L/h (3.1–10.7), respectively.

**Conclusion:**

This study demonstrates that isavuconazole trough concentrations are often maintained above 1 mg/L despite patients being on concomitant rifabutin. However, therapeutic drug monitoring is mandatory in this setting. Further research is warranted to confirm these results.

## 1 Introduction

In 1984, the interaction between rifamycins and azole antifungals was first described in a patient in whom decreased serum concentrations of rifampin and ketoconazole were identified following co‐administration of these drugs [1]. Mycobacterium has been shown to be a risk factor for the development of infection due to *Aspergillus* and therefore concomitant treatment can be necessary [2, 3]. Rifamycins, such as rifampin and rifabutin, are typically used for their activity against mycobacterium while isavuconazole, voriconazole and posaconazole have been shown in randomised trials to be effective in the treatment of *Aspergillus* spp. [4]. Isavuconazole, the newest azole antifungal, represents an attractive therapeutic and prophylactic option given its broader spectrum of activity against Mucorales with a favourable adverse effect profile compared to voriconazole [4].

Since rifabutin has less severe drug interactions, it is preferred over rifampin when administered concomitantly with azole antifungals. Pharmacokinetic data for posaconazole and voriconazole with rifabutin remain limited, and the effect of rifabutin on isavuconazole pharmacokinetics has been limited to two prior case reports [5, 6, 7].

## 2 Methods

This was a single‐centre, retrospective study of hospitalised patients who received concomitant isavuconazole and rifabutin prior to plasma isavuconazole therapeutic drug monitoring between January 2018 and March 2025. Patients were excluded if they required renal replacement therapy and if no therapeutic drug monitoring was performed throughout the duration of treatment course. Isavuconazole was administered at a standard dose of isavuconazonium sulphate 372 mg every 8 h for six doses followed by 372 mg every 24 h. The intravenous formulation of isavuconazole was administered over a 1‐h infusion. Patients were considered critically ill if they required vasopressor support or invasive mechanical ventilation. Isavuconazole plasma concentrations were determined by a validated high pressure liquid chromatography‐UV (HPLC‐fluorescence) assay technique [8].

Pharmacokinetic analyses were performed using InsightRX, leveraging a previously published population pharmacokinetic model [9, 10]. Model fitting was conducted using a maximum a posteriori Bayesian approach. Because there are no existing models to simulate isavuconazole induction by rifabutin, the prior was intentionally flattened to better accommodate the ongoing induction process [9, 10]. A linear gradient weighting scheme was applied during fitting. Isavuconazole plasma concentrations were used to generate all pharmacokinetic parameters. Corrected 24‐h steady‐state concentrations were considered therapeutic if the isavuconazole level was > 1 mg/L [11]. Population estimates in the absence of rifabutin were used for comparison. This study was approved by the University of Pittsburgh Medical Center institutional review board (STUDY22070065).

## 3 Results

### 3.1 Overview

Of the nine patients who received concomitant rifabutin and isavuconazole, two patients were excluded given an isavuconazole level was not obtained. Clinical data of the seven included patients is summarised in Table 1. The median age (range) was 53 years (33–67), 71% (5/7) were solid organ transplant recipients, and no patients had underlying cirrhosis. The indications for isavuconazole use included empiric treatment (Patients 1, 2 and 7), prophylactic use (Patient 3) and *Aspergillus* treatment (Patients 4, 5 and 6). Indications for rifabutin included infection due to *Mycobacterium avium* complex in all patients except for Patient 2 who was receiving treatment for *Mycobacterium tuberculosis* . The median (range) duration of concomitant rifabutin and isavuconazole prior to first isavuconazole therapeutic drug monitoring was 133 h (44–184).

**TABLE 1:** Summary of patients who received concomitant isavuconazole (ISA) and rifabutin.

Patient	Age/Gender	Ethnicity	Weight (kg)	Transplant^a^	Cystic fibrosis	Albumin (g/dL)	Critical illness	Enteral versus intravenous	Rifabutin dose	Time from last ISA dose to level	ISA level (mg/L)^b^
1	69/F	Caucasian	44	Renal	No	2.8	No	Intravenous	300 mg daily	69 h	1.4
2^c^	49/M	African American	66	Lung	Yes	2.4	Yes	Intravenous	300 mg daily	20 h	4.2
3	64/F	Caucasian	61	Lung	No	1.9	No	Enteral	150 mg daily	24 h	1.2
4	49/M	Caucasian	59	Lung	No	—	No	Enteral	300 mg daily	24 h	1.9
5^e^	74/F	Caucasian	58	Lung	No	4.2	No	Enteral	300 mg daily	49 h	2.1
6^d^	53/F	Caucasian	58	None	No	—	No	Intravenous	300 mg daily	26 h	0.7
7^e^	33/F	African American	42	None	No	2	Yes	Enteral	300 mg daily	11 h	5.9

^a^ Patients 1–4 received tacrolimus while Patient 5 received cyclosporine.

^b^ ISA level of patients receiving concomitant rifabutin.

^c^ The patient was noted to have rifabutin levels of 0.31 and 0.32 μg/mL at 3 and 7 h, respectively, while on ISA.

^d^ ISA level drawn 21 h after the last dose in the absence of a rifamycin was 1.5 (mg/L); ISA level drawn 22 h after the last dose with concomitant rifampin was 0.4 (mg/L).

^e^ Patients 5 and 7 experienced 90‐day mortality.

### 3.2 Pharmacokinetic Analysis

The median (range) time from last isavuconazole dose administration to isavuconazole level obtainment was 24 h (22–38) and the median isavuconazole (range) level without time correction was 1.9 mg/L (1.3–3.2). The median (range) corrected 24‐h steady‐state isavuconazole level was 2.7 mg/L (0.7–3.7) and 86% (6/7) had a therapeutic level, as noted in Table 2. The median (range) area under the curve (AUC 24), half‐life (*T*_½_) and clearance (Cl) were 85 mg/L h (33–112), 13.1 h (6.8–17.8) and 4 L/h (3.1–10.7), respectively. The median individual estimate for Cl was 1.6‐fold higher than the population estimate at 2.4 L/h. Conversely, the population estimate for *T*_½_ was 1.5‐fold higher than the individual estimate. No patients had the isavuconazole dose adjusted following level obtainment.

**TABLE 2:** Pharmacokinetic analysis of patients who received concomitant isavuconazole (ISA) and rifabutin.

Patient	Individual isavuconazole pharmacokinetic calculations using observed level				Estimated population isavuconazole parameters^b^	
	24‐h steady‐state level (mg/L)	AUC 24 (mg/L h)	*T*_½_ (h)	Cl (L/h)	*T*_½_ (h)	Cl (L/h)
1	2.7	90	15	3.8	22.1	2.4
2	3.7	112	17.8	3.1	22.4	2.4
3	1.1	45	8.2	7.8	19.3	2.4
4	1.8	65	10.1	5.37	18.9	2.4
5	2.9	88	13.1	4	19.4	2.4
6^a^	0.7	33	6.8	10.7	19.4	2.4
7	2.7	85	13.1	3.9	19	2.4
Median (range)	2.7 (0.7–3.7)	85 (33–112)	13.1 (6.8–17.8)	4 (3.1–10.7)	19.4 (18.9–22.4)	2.4 (2.4–2.4)

^a^ On rifampin, the 24 h steady‐state ISA level, AUC_24_, half‐life and clearance were 0.3 mg/L, 20 mg/L h, 5.2 h and 17.6 L/h, respectively. In the absence of a rifamycin, the 24 h steady‐state ISA level, AUC_24_, half‐life and clearance were 1.4 mg/L, 56 mg/L h, 8.9 h and 6.3 L/h, respectively.

^b^ Calculations are predicted using insightRx for population pharmacokinetics in the absence of rifabutin.

In addition to therapeutic drug monitoring of isavuconazole while on rifabutin, Patient 6 had therapeutic drug monitoring of isavuconazole while on rifampin and in the absence of a rifamycin. Patient 6 was initially started on rifampin, then transitioned to rifabutin. She ultimately experienced nausea and therefore rifabutin was discontinued. For Patient 6, the corrected 24‐h steady‐state isavuconazole level while on rifampin, rifabutin and no rifamycin was 0.3, 0.7 and 1.4 mg/L, respectively.

### 3.3 Clinical Outcomes

A breakthrough invasive mould infection was diagnosed in 14% (1/7) of patients. Patient 4 was diagnosed with a breakthrough culture documented invasive *A. fumigatus* infection 24 days after the initial isavuconazole level of 1.9. While maintained on 300 mg daily of rifabutin, a repeat isavuconazole level drawn 23 h after the last dose of isavuconazole was obtained, resulting in 1.5 mg/L. The patient was then transitioned to posaconazole following the breakthrough infection; however, the cause of the breakthrough infection was thought to be secondary to immunodeficiency and not subtherapeutic isavuconazole levels. Of the included patients, 29% (2/7) experienced 90‐day mortality.

## 4 Discussion

To our knowledge, this is the largest case series to evaluate the effect of rifabutin on isavuconazole exposure. Two previous cases of patients on rifabutin and isavuconazole have reported isavuconazole levels of 0.9 and 4.5 mg/L, suggesting interpatient variability [7]. It has been hypothesised that this pharmacokinetic variability could be a result of alterations in the CYP3A‐genotype which may explain the heterogenous pharmacokinetic parameters in the current case series [7].

The mechanisms of this interaction are rifabutin‐mediated induction of CYP3A4 metabolism of isavuconazole, leading to reduced isavuconazole concentrations. In a pharmacokinetic study of 24 healthy volunteers, rifampin, a stronger CYP3A4 inducer than rifabutin, decreased the isavuconazole AUC by 97% [12]. These results are comparable to the observed pharmacokinetics of Patient 6 in which the 24‐h steady‐state isavuconazole trough was nearly five times higher in the presence of rifampin compared to the absence of any rifamycin.

In the SECURE trial, the median steady‐state isavuconazole trough concentration and steady‐state AUC were 3.2 mg/L and 90 mg/L h, respectively [13]. Compared to these values, we observed reduced isavuconazole exposure which may have been due to rifabutin. Nevertheless, 86% of patients in the present study still had a steady state 24‐h isavuconazole level > 1 mg/L, suggesting the interaction may be manageable with therapeutic drug monitoring and subsequent dose adjustment. Although, no clear pharmacokinetic targets exist, consensus guidelines have advised maintaining isavuconazole trough concentrations > 1 mg/L since 90% of patients in the SECURE trial were found to have trough concentrations > 1 mg/L [11, 14, 15]. However, no concentration dependent relationship has been observed for efficacy [14, 15].

While the strength of this study is in its pharmacokinetic analysis, several limitations of this study are recognised. First, only seven patients were able to be included. However, given this study was performed over a 6‐year period, larger real‐world studies may be difficult to conduct. Second, only Patient 2 had rifabutin therapeutic drug monitoring and isavuconazole‐mediated inhibition of CYP3A4 may lead to increased rifabutin concentrations. Notably, this patient had therapeutic levels of 0.31 and 0.32 μg/mL at 3 and 7 h, respectively, and only Patient 6 discontinued rifabutin due to intolerance. Until further research can elucidate isavuconazole's impact on rifabutin pharmacokinetics, therapeutic drug monitoring for rifabutin should be considered mandatory when administered concomitantly with isavuconazole. Third, only a single isavuconazole level had been obtained for each patient while on rifabutin. Current guidelines recommend obtaining an isavuconazole level after Day 5 of therapy. Therefore, it is possible that some levels were not at steady state considering isavuconazole levels were obtained after a median (range) of 133 h (44–184) of concomitant rifabutin and isavuconazole [11]. Fourth, it is recognised that the median simulated isavuconazole *T*_½_ was reported to be 19 h whereas an isavuconazole *T*_½_ of 66 h has been observed in a similar population [8]. Finally, this real‐world study included a heterogenous patient population which included several solid organ transplant and critically ill patients which may confound pharmacokinetic calculations.

This study demonstrates that isavuconazole trough concentrations are often maintained above 1 mg/L despite patients being on concomitant rifabutin. However, therapeutic drug monitoring is mandatory in this setting. Further research is warranted to confirm these results.

## Funding

This work was not directly supported by any funding agencies. This study was performed as part of our routine work.

## Data Availability

The data that support the findings of this study are available on request from the corresponding author. The data are not publicly available due to privacy or ethical restrictions.
